# Inhibition of GLI Transcriptional Activity and Prostate Cancer Cell Growth and Proliferation by DAX1

**DOI:** 10.3390/cimb45070339

**Published:** 2023-06-27

**Authors:** Sung Pyo Hong, Kil Won Kim, Soon Kil Ahn

**Affiliations:** Institute for New Drug Development, Division of Life Sciences, Incheon National University, Incheon 22012, Republic of Korea

**Keywords:** DAX1, GLI transcription factor, hedgehog signaling, prostate cancer

## Abstract

The Hedgehog (Hh) signaling pathway plays an essential role in the initiation and progression of prostate cancer. This is mediated by transcriptional factors belonging to the GLI (glioma-associated oncogene) family, which regulate downstream targets to drive prostate cancer progression. The activity of GLI proteins is tightly controlled by a range of mechanisms, including molecular interactions and post-translational modifications. In particular, mitogenic and oncogenic signaling pathways have been shown to regulate GLI protein activity independently of upstream Hh pathway signaling. Identifying GLI protein regulators is critical for the development of targeted therapies that can improve patient outcomes. This study aimed to identify a novel protein that directly regulates the activity of GLI transcription factors in prostate cancer. We performed gene expression, cellular analyses, and reporter assays to demonstrate that DAX1 (dosage-sensitive sex reversal adrenal hypoplasia congenital critical region on X chromosome, gene 1) interacts with GLI1 and GLI2, the master regulators of Hh signaling. Interestingly, DAX1 overexpression significantly inhibited Hh signaling by reducing GLI1 and GLI2 activity, prostate cancer cell proliferation, and viability. Our results shed light on a novel regulatory mechanism of Hh signaling in prostate cancer cells. The interaction between DAX1 and GLI transcription factors provides insight into the complex regulation of Hh signaling in prostate cancer. Given the importance of Hh signaling in prostate cancer progression, targeting DAX1–GLI interactions may represent a promising therapeutic approach against prostate cancer. Overall, this study provides new insights into the regulation of the Hh pathway and its role in prostate cancer progression. The findings suggest that DAX1 could serve as a potential therapeutic target for the treatment of prostate cancer.

## 1. Introduction

Prostate cancer mortality rates are higher in low/middle-income countries such as African countries, even though high-income countries have high prostate cancer incidence rates. Androgen-deprivation therapy is the standard treatment for patients with prostate cancer. However, prostate cancer eventually develops resistance to hormone therapy and progresses to fatal metastatic castration-resistant prostate cancer [1]. Therefore, there is a need to identify an alternative target to block the activity of a key driver of androgen-independent or castration-resistant prostate cancer.

Hedgehog (Hh) signaling plays an important role in the growth and progression to a more aggressive phenotype of prostate cancer [2]. In vertebrates, the Hh signaling pathway is initiated by one of the three ligands: Sonic, Indian, and Desert hedgehog [3]. The primary receptor of these ligands is the transmembrane protein Patched 1 (PTCH1) [4,5]. Without ligand stimulation, PTCH1 inhibits SMOOTHENED (SMO). However, upon ligand stimulation, PTCH1 relieves SMO inhibition, and the signal is transduced to the zinc-finger transcription factors GLI1, GLI2, and GLI3 [6]. The GLI transcription factor family is a subgroup of the Krüppel transcription factor family. These transcription factors share a DNA-binding zinc-finger domain that is highly conserved [7,8]. GLI1-3 transcription factors contain a central five-finger domain in each protein. Within this domain, fingers 2–5 are responsible for recognizing and binding to the GACCACCCA motif present in the DNA sequence. During this process, these fingers contact the major groove of the DNA and wrap around it. It is believed that the zinc fingers within GLI transcription factors may also facilitate protein–protein interactions [7].

GLI transcription factors control the expression of Hh target genes involved in critical cellular activities, such as cell cycle regulation, proliferation, migration, and self-renewal [4]. GLI1 and GLI2 serve as the primary transcriptional activators, while GLI3 functions primarily as a transcriptional repressor depending on the cell context [9]. GLI proteins influence the expression of both GLI1 and PTCH1, thus establishing a feedback loop that either stimulates or inhibits the Hh response. Additionally, a noncanonical activation of GLI proteins is reported, independent of Hh signaling [10,11,12]. Therefore, GLI protein activation should be considered regardless of upstream signaling cascades.

Furthermore, GLI1 and GLI2 are the main transcriptional effectors, and their constitutive activation is the most important risk factor for cancer development and progression. Thus, GLI proteins are emerging as important targets for the development of new anticancer drugs [13]. However, only a few GLI regulators have been identified, owing to the lack of structural information related to GLI activity. Therefore, this study focused on the identification of the proteins that regulate GLI proteins, the final effectors of the Hh signaling, which may help overcome anti-SMO resistance. In addition, it is important to note that most of the small molecule pathway inhibitors generated from these programs specifically focus on inhibiting SMO and do not exhibit efficacy against Hh/GLI-dependent cancers that develop from mutations occurring downstream of SMO or from GLI1/GLI2 overexpression. Considering this, several researchers have turned their attention downstream in the conventional Hh/GLI1 signaling pathway to discover inhibitors that directly target GLI1/GLI2 [14].

DAX1 (dosage-sensitive sex reversal adrenal hypoplasia critical region on the X chromosome, gene 1) is an orphan nuclear receptor encoded by the *NR0B1* gene (nuclear receptor subfamily 0, group B, member 1). DAX-1 has a conserved ligand-binding domain and a potent transcriptional repressor domain in the C-terminal region, but it lacks a classical DNA-binding domain. Its unique structure includes twelve helices in the carboxyl-terminal region, similar to those found in other nuclear receptors, and an amino-terminal region containing 66–67 amino acids with LXXLL motifs [15]. DAX1 contains three LXXLL motifs and is thus considered an LXXLL-containing co-repressor. These motifs are important for interactions of DAX1 with other proteins in the nucleus, particularly with other nuclear receptors and co-regulators, that help DAX1 regulate the expression of target genes [16]. DAX1 has the ability to influence the activity of different nuclear receptors, including steroidogenic factor-1, estrogen receptor, progesterone receptor, and androgen receptor. Thus, DAX1 can affect how these receptors function in the nucleus, altering their ability to bind to DNA and regulate the expression of target genes. The ability of DAX1 to modulate the activity of these receptors highlights its important role as a co-regulator protein in the regulation of gene expression. However, the ligand that can switch its transcriptional characteristics from a repressor to an activator remains unknown [17,18]. DAX1 primarily inhibits the transcriptional activity of multiple transcription factors and nuclear receptors through mechanisms that involve both interaction with the coactivator binding surface of the transcription factors or interference with the dimerization of nuclear receptors [18]. DAX1 is expressed in several cancers, although its expression pattern in tumor growth has shown discrepancy among different types of tumors. In addition, DAX1 expression in normal and tumor tissues of the prostate is controversial [19]. The exact function of DAX1 in prostate cancer is still unclear and needs to be further investigated.

In this study, we investigated the role of DAX1 in regulating the Hh signaling pathway in prostate cancer cells. Our findings provide new insights into the complex regulation of the Hh pathway in prostate cancer and suggest a potential therapeutic target for Hh-driven tumors. Specifically, we demonstrated that DAX1 overexpression significantly inhibits Hh signaling by reducing the activity of GLI1 and GLI2, master regulators of the Hh pathway. These results highlight the novel role of DAX1 in regulating Hh signaling in prostate cancer cells and provide a foundation for future studies aimed at understanding the mechanism of the DAX1-mediated inhibition of GLI activity. Our study has important implications for the development of targeted therapies for Hh-driven tumors.

## 2. Materials and Methods

### 2.1. Plasmid Constructs and Reagents

pBluescrpit-Gli1 was kindly provided from Dr. Kenneth W. Kinzler (Johns Hopkins University, Baltimore, MD, USA). GLI1 fragments were subcloned into plasmids pcDNA3/GFP and pcDNA3/HA. The 8×Gli-bs-luc reporter (eight copies of the GLI-binding sites connected to the luciferase reporter) was kindly provided by Dr. Hiroshi Sasaki (Osaka University, Osaka, Japan). pcDNA3/FLAG was constructed as described previously [20]. DAX1 fragments were subcloned into pcDNA3/RFP and pcDNA3/FLAG. The PTCH1-luc reporter constructs (containing the *Ptch1* promoters fused to the luciferase reporter, respectively), the GLI1-luc reporter, and pcDNA3.1/His-GLI2 were a kind gift from Dr. Fritz Aberger (University of Salzburg, Salzburg, Austria). GLI2 fragments were subcloned into plasmids pCS2-GFP and pcDNA3-6Myc. SMO agonist (SAG) was purchased from Sigma-Aldrich (Seoul, Republic of Korea).

### 2.2. Cell Lines and Cell Culture

HEK293, PC3, and DU145 cell lines were purchased from the Korean Cell Line Bank (Republic of Korea). ALL cell lines were maintained in DMEM or RPMI supplemented with 10% FBS and 1% penicillin–streptomycin in an incubator at 37 °C and 5% CO_2_.

### 2.3. Cell Growth Assay

The CellTiter 96 Non-Radioactive Cell Proliferation Assay kit (Promega, Republic of Korea) was used following the manufacturer’s instructions. Briefly, cell suspension (100 μL) was dispensed into a 96-well plate (5 × 10^3^ cells/well; Spl life sciences (Seoul, Republic of Korea), Republic of Korea) and incubated at 37 °C. Then, dye solution was added (15 μL/well), the cells were incubated at 37 °C for up to 4 h, and the solubilization solution/stop mix was added to every well. The absorbance at 570 nm was detected with a microplate reader (Molecular Devices; Silicon Valley, CA, USA). The relative cell proliferation rate was calculated as the following ratio: (optical density of experimental wells/optical density of control wells) × 100.

### 2.4. Western Blot Analysis and Co-Immunoprecipitation

Proteins from total lysates or immune-precipitated complexes were separated by SDS-PAGE and blotted onto PVDF membranes using a Bio-Rad Mini Trans Blot Electrophoretic Transfer cell. The primary antibodies used are as follows: anti-DAX1 (cell signaling technology; Danvers, MA, USA), anti-GLI1 (cell signaling technology; Danvers, MA, USA), anti-GLI2 (Abcam; Cambridge, UK), anti-PTCH1 (Abcam; Cambridge, UK), anti-FLAG (Sigma-Aldrich; Seoul, Republic of Korea), anti-HA (Sigma–Aldrich; Seoul, Republic of Korea), anti-c-Myc (Sigma–Aldrich; Seoul, Republic of Korea), and anti-SMO (Abcam; Cambridge, UK). Anti-rabbit, anti-mouse, and anti-rat IgG horseradish peroxidase linked secondary antibodies were obtained from Santa Cruz biotechnology. M2 Flag-agarose was purchased from Sigma-Aldrich and used for co-immunoprecipitation studies. A total of 2 mg of cell lysate was used for endogenous co-immunoprecipitation assay. As a negative control, normal IgG antibodies were used. Normal IgG for IP-DAX1 is rabbit-IgG. Immunoprecipitated proteins were resolved by SDS-PAGE followed by Western blot analysis. Antibodies used for endogenous co-immunoprecipitation are as follows: anti-DAX1 (cell signaling technology; Danvers, MA, USA), normal IgG (Santa Cruz biotechnology; Dallas, TX, USA). Antigen–antibody complexes were visualized using a chemiluminescent substrate (Millipore; Billerica, Massachusetts, USA) and detected using the ChemiDoc MP imaging system (Bio-Rad Laboratories; Berkeley, CA, USA).

### 2.5. Quantitative PCR (qRT-PCR)

Total RNA from cells was extracted using an RNeasy Plus mini kit (Qiagen; Wiesbaden, Germany). cDNAs were produced using a Superscript II enzyme (Invitrogen; Waltham, MA, USA). The cDNA synthesis protocol typically involves adding Superscript II enzyme to a reaction mixture containing RNA template, oligo(dT) primers, and other reaction components. First, the RNA template is denatured by heating at 70 °C for 5 min and then cooled on ice. Then, the reaction mixture containing the RNA template, oligo(dT) primers, dNTPs, buffer, and Superscript II enzyme is added to the RNA template and incubated at 42 °C for 60 min to allow for the reverse transcription of the RNA into cDNA. After the incubation, the reaction is terminated by heating at 70 °C for 15 min. The resulting cDNA can then be analyzed using a SYBR green PCR kit and Thermal Cycler DICE Real Time System TP800 (Takara; Kusatsu, Japan). All data were normalized to L32.

### 2.6. SiRNA Experiments

Control scrambled siRNA and DAX1 siRNA, that contained four distinct siRNA species targeting different sequences of the DAX1 transcript, were purchased from Dharmacon. DU145 or PC3 cells were transfected with siRNA using DharmaFECT-1, a transfection reagent manufactured by Dharmacon. The siRNA is first mixed with DharmaFECT-1 in Opti-MEM, a reduced-serum media, and then added to the cells. The cells are then incubated with the siRNA-DharmaFECT-1 mixture for a certain period of time, typically 72 h, to allow for effective transfection and knockdown of the target gene.

### 2.7. Transient Transfections and Luciferase Assays

The transient transfections were performed using TransIT-LT1 (Mirus; Houston, TX, USA) according to the manufacturer’s instructions. For luciferase reporter assays, HEK293 cells were seeded and transfected using the indicated plasmids. Cells were harvested 48 h after transfection. Using the β-galactosidase activity, the luciferase activity was corrected for transfection efficiency. All tests were conducted in at least triplicate.

### 2.8. Cell Cycle Assay

The cells (1 × 10^6^ cells per sample) were fixed with 70% ethanol and stained with 40 μg/mL propidium iodide (Becton Dickinson; Franklin Lakes, NJ, USA) for 10 min. The distribution of the cell cycle was evaluated by measuring DNA content with the Gallios flow cytometer and Kaluza software (Beckman Coulter; Brea, CA, USA). At least 1 × 10^4^ cells per data point were evaluated.

### 2.9. Colony Formation Assay

Cells were plated in six-well plates (900 cells/well). After 14 days, the cells were treated with methanol fixation and then stained with Crystal Violet (Sigma-Aldrich). Colonies were counted and imaged by the Canon 550D camera.

### 2.10. MTT Assay

The CellTiter 96 Nonradioactive Cell Proliferation Assay kit (Promega; Fitchburg, WI, USA) was used following the manufacturer’s instructions. In brief, 100 μL of the cell mixture containing 5 × 10^3^ cells was added to each well of a 96-well plate (SPL Life Sciences) and left to incubate overnight. At the end of the incubation time, 15 μL of the dye was added to each well, and the cells were further incubated for up to 4 h at 37 °C. The solubilization solution/stop mix was applied to each well following incubation. Absorbance at 570 nm was measured with a microplate reader (Molecular Devices). The relative rate of cell proliferation was defined as the following ratio: (optical density of experimental wells/optical density of control wells) × 100. IC_50_ values were generated using a 12-point curve fitted with Prism 8 (GraphPad; San Diego, CA, USA)).

### 2.11. Lentivirus and Establishment of Stable DU145 Cell Lines Overexpressing DAX1 or Control

pMDLg/pRRE, pMD2.G, and pRSV-Rev were purchased from Addgene (plasmids #12251, #12259, and #12253). These plasmids were a gift from Didier Trono (University of Geneva Medical School, Geneva, Switzerland). Recombinant lentiviruses were produced by co-transfecting HEK293 cells with the target plasmid, packaging plasmid, and envelope plasmid and then collected from the virus-containing cell culture media. Next, DU145 or PC3 cells were infected with the harvested lentiviruses. Stable cell lines expressing the targeted lentiviral vectors were selected using puromycin (Thermo Fisher Scientific; Seoul, Republic of Korea).

### 2.12. Fluorescence Microscopy of Living Cells

Fluorescence microscopy was performed on HEK293 cells transfected with pcDNA3-GFP-GLI1, pCS2-GFP-GLI2, and pcDNA3-RFP-DAX1 using the TransIT-LT1 Transfection Reagent (Mirus; Houston, TX, USA). Following transfection, the cells were incubated for 24 h. The nuclei of the cells were stained with Hoechst dye for 10 min at 37 °C and visualized with a Carl Zeiss (Oberkochen, Germany) Axiovert 200 M microscope.

### 2.13. Co-Immunoprecipitation Assay

After 48 h of transfection, cells were harvested and washed with ice-cold PBS. The cells were then lysed in lysis buffer (50 mM Tris-HCl pH 7.4, 150 mM NaCl, 1 mM EDTA, 1% Triton X-100, 1 mM PMSF) and incubated on ice for 30 min. The lysates were cleared by centrifugation at 14,000 rpm for 10 min at 4 °C. The cleared lysates were then mixed with 20 μL of M2 (flag)-agarose beads and rotated for 16 h at 4 °C to immunoprecipitate the DAX1 protein. The bound proteins were eluted from the beads by boiling in SDS sample buffer (50 mM Tris-HCl pH 6.8, 2% SDS, 10% glycerol, 100 mM DTT, 0.1% bromophenol blue) and separated by SDS-PAGE on 10% gels. The proteins were then transferred to polyvinylidene difluoride membranes (Millipore Corp., Bedford, MA, USA) and probed with anti-HA, anti-MYC, or anti-FLAG antibody. The membranes were developed using an ECL kit (GE Healthcare; Chicago, IL, USA) and the signals were detected using a ChemiDoc MP Imaging System (Bio-Rad Laboratories; Berkeley, CA, USA).

### 2.14. Immunofluorescence Staining

After the incubation with SAG, PC3 cells were fixed using 4% paraformaldehyde (Sigma-Aldrich, Seoul, Republic of Korea). This was followed by the permeabilization of the cells using 0.1% Triton X-100 (Sigma-Aldrich, USA) for 10 min. The fixed and permeabilized cells were then incubated in a blocking solution containing 5% skim milk for 1 h. PC3 cells were immunostained with primary anti-Ki67 antibody from Santa Cruz (Dallas, TX, USA) and secondary goat anti-mouse antibody from Invitrogen (USA). Mounting of the stained cells was performed with ProLong Gold Antifade Mountant with DAPI (Invitrogen; Waltham, MA, USA). Using the Agilent BioTek Cytation 1 device (USA), fluorescent images of the samples were acquired. The Gen5 program was used to extract relevant data from the images and conduct quantitative analysis.

### 2.15. Statistical Analysis

The data are presented as the mean and standard error of the mean (SEM). Group comparisons were analyzed through one-way or two-way ANOVA. A significance level of *p* ≤ 0.05 was considered statistically significant. Correlation analysis was performed using GraphPad Prism. The significance of the correlation was demonstrated by a *p* value.

## 3. Results

### 3.1. DAX1 Inhibits the Hh Pathway

**Figure 1:**
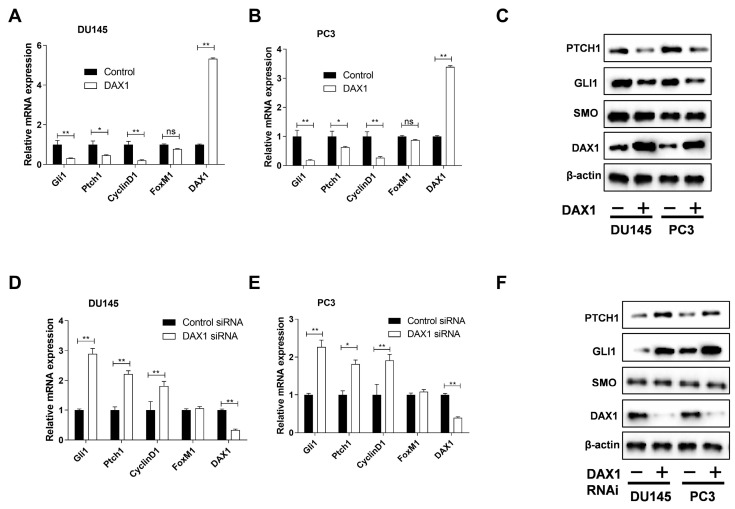

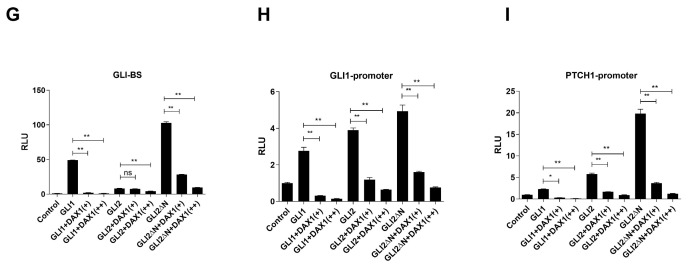
DAX1 inhibits the Hedgehog pathway. (**A**–**C**) The mRNA and protein levels of DAX1 and Hedgehog (Hh) target gene expression levels in DU145 and PC3 cells transduced with lenti-DAX1 or backbone vector control. Relative gene expression levels normalized to respective control levels are shown. A one-way ANOVA with Bonferroni posttest was used to compare the DAX1 overexpression effect with the control. (**D**–**F**) The mRNA and protein levels of the indicated genes in DU145 and PC3 cells treated with control or DAX1 siRNA. The gene expression was normalized and statistical analysis was performed as described above. (**G**) A construct linking the eight repeats of GLI-binding sites to a luciferase reporter (8×GLI-bs-luc reporter) was used as a surrogate measurement of the GLI-dependent transcription. All measured luciferase activities were normalized to the empty vector. HEK293 cells were co-transfected with the 8×GLI-bs-luc reporter and the plasmid encoding GLI1, GLI2, or GLI2ΔN expression vectors for DAX1 or control vector. (**H**,**I**) HEK293 cells were transiently co-transfected with *Gli1* or *Ptch1* promoter luciferase constructs as reporters and GLI1, GLI2, or GLI2 lacking the N-terminal repression domain (GLI2ΔN) expression vectors for DAX1 or empty vector. A two-way ANOVA with Bonferroni posttest was used for the statistical analyses. The experiment was repeated thrice. Data represent mean ± SEM; * *p* ≤ 0.05, ** *p* ≤ 0.01; ns, not significant.

To evaluate the role of DAX1 in the Hh pathway, we performed DAX1-overexpression and siRNA-knockdown experiments in the prostate cancer cell lines DU145 and PC3. DAX1 overexpression by retroviral delivery significantly inhibited the Hh pathway and decreased the expression of downstream genes *Gli1*, *Ptch1*, and *CCND1* in both PC3 and DU145 cell lines. In addition to reducing the mRNA expression of hedgehog target genes, our findings demonstrated that DAX1 also caused a decrease in the protein levels of Gli1 and Ptch1. However, DAX1 did not induce any alterations in the levels of SMO protein and FOXM1 mRNA (Figure 1A–C). Conversely, upon DAX1 knockdown, we observed the activation of the Hh pathway, as evidenced by increased levels of both Gli1 and Ptch1 mRNA and protein (Figure 1D–F). Furthermore, our results demonstrate that DAX1 knockdown had no effect on the abundance of SMO protein. To investigate whether DAX1 regulates GLI1 and GLI2 activity, we used a luciferase reporter vector for a GLI-binding site (GLI-bs-luc). The transcriptional activity of GLI1 and GLI2 was notably suppressed by DAX1 (Figure 1G). In particular, a constitutively active form of GLI2 lacking the N-terminal repression domain (GLI2ΔN) was more suppressed than the full-length GLI2 with the N-terminal repressor domain. GLI protein-mediated GLI1 induction initiates the positive feedback loop of the Hh pathway, while the GLI protein-mediated regulation of PTCH1 initiates the negative feedback loop [6]. However, *Ptch1* is a well-characterized direct target gene of GLI and its upregulated expression is indicative of Hh pathway activation [21]. To assess whether DAX1 could repress the induction of the GLI protein target genes, we conducted a luciferase assay using a reporter vector containing the promoters of *Gli1* and *Ptch1* (Figure 1H,I). The results indicate that DAX1 markedly reduced the transcriptional activation of GLI proteins.

### 3.2. DAX1 Interacts with GLI1 and GLI2

**Figure 2:**
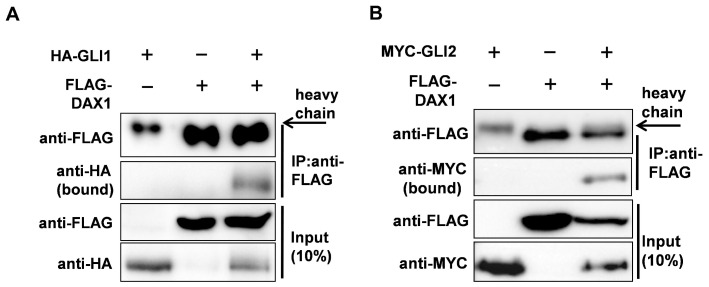

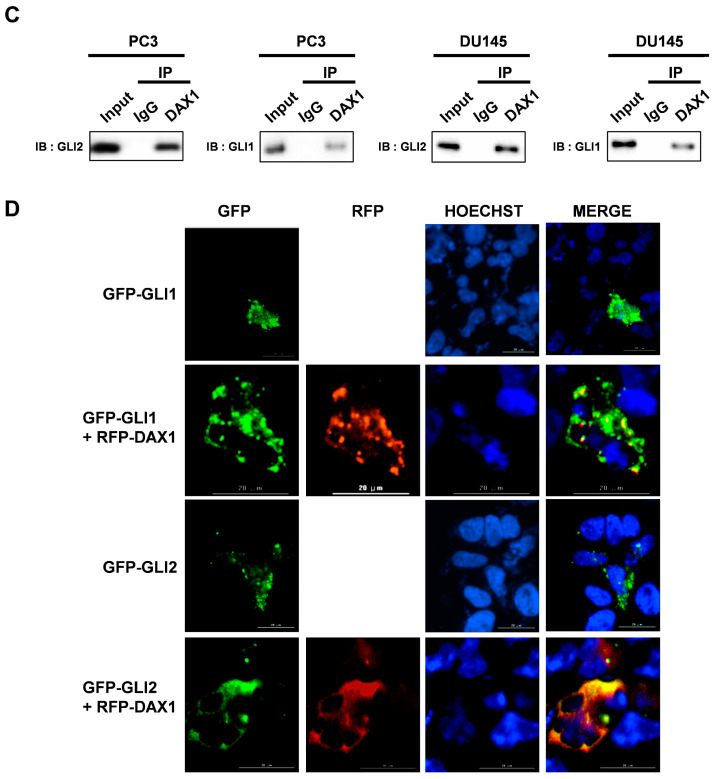
DAX1 interacts with GLI1 and GLI2. (**A**,**B**) Co-immunoprecipitation assay showing the interaction between DAX1 and GLI proteins. Flag-tagged DAX1 expression vector was co-transfected with empty vector, HA-tagged GLI1 expression vector, or MYC-tagged GLI2 expression vector into HEK293 cells for 48 h before being harvested. Immunoprecipitation was performed with either M2 FLAG-agarose and blotted with antibodies against Flag, HA, or MYC. (**C**) Co-immunoprecipitation of endogenous DAX1-GLI1 or DAX1-GLI2. DAX1 antibodies were employed for the immunoprecipitation of DAX1 protein from whole cell extracts of PC3 or DU145 cells. Following the immunoprecipitation, Western blotting was performed to probe the samples for GLI1 or GLI2 proteins, respectively. (**D**) GFP–GlI1 or GFP–GlI2 plasmid was co-transfected with RFP–DAX1 plasmids into HEK293 cells. After a 36 h transfection, the cells were counterstained with Hoechst to label nuclei and assessed using fluorescence microscopy.

To determine whether the DAX1-induced repression of GLI transcriptional activity was mediated by the GLIs–DAX1 interaction, a co-immunoprecipitation assay was performed. Flag-DAX1 proteins were co-immunoprecipitated with HA–GLI1, but not with the control HA protein, as shown by Western blot analysis (Figure 2A). Additionally, Flag-tagged DAX1 proteins also interacted with MYC-tagged GLI2 (Figure 2B). To investigate the potential interaction between endogenous DAX1 and GLI proteins in prostate cancer cells, co-immunoprecipitation assays were conducted in PC3 and DU145 cell lines. The co-immunoprecipitation assay revealed that when the DAX1 antibody was precipitated with prostate cancer cell lysates, it bound to GLI proteins (Figure 2C). As GLI proteins are nuclear-cytoplasmic shuttling proteins [22], GLI proteins can be found in the cytoplasm and nucleus depending on the context [23,24]. To determine whether DAX1 was co-localized with GLI proteins, GFP-tagged GLI1 or GFP-tagged GLI2 were co-transfected with RFP-tagged DAX1 into cells and the subcellular localization of each protein was compared. RFP-DAX1 co-localized with GFP-GLI1 and GFP-GLI2 (Figure 2D).

### 3.3. DAX1 Induces G1/S Cell Cycle Arrest

**Figure 3:**
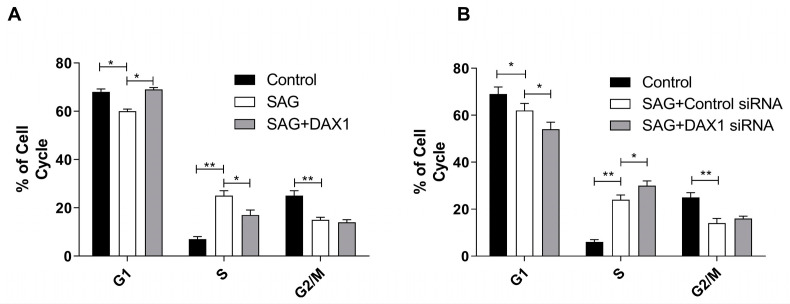
DAX1 induces G1/S cell cycle arrest. (**A**) Flow cytometric cell cycle analysis of DU145 cells transduced with lenti-DAX1 or the backbone vector control. DU145 cells were incubated with 30 nM SAG for 60 h. (**B**) Flow cytometric cell cycle analysis of DU145 cells treated with control or DAX1 siRNA. DU145 cells were incubated with 30 nM SAG for 60 h. The percentages of cells in different phases of cell cycle are represented by a bar diagram. Propidium iodide fluorescence was measured using a flow cytometer with an FL-2 filter. A two-way ANOVA with Bonferroni posttest was used for the statistical analyses. The experiment was conducted three times. Data represent mean ± SEM; * *p* ≤ 0.05, ** *p* ≤ 0.01.

Cell proliferation is regulated by the progression of the cell cycle, which consists of G1, S, G2, and M phases. Cyclin-dependent kinases (CDKs) associated with cyclins are responsible for the cell-cycle machinery [6]. During the G1 phase of the cell cycle, CDK4 and CDK6 are guided by D-type cyclins. D-type cyclins are induced by the Hh pathway due to GLI-binding sites within the *CCND1* promoter regions [25]. Our findings indicate that DAX1 downregulated the expression of CCND1 in prostate cancer cell lines (Figure 1A,B). To investigate the relationship between DAX1 expression and the cell cycle progression, the prostate cancer cell line DU145 was transduced to stably overexpress DAX1 or the vector control. To elucidate the role of DAX1 in the Hh pathway, DU145 cells were subjected to treatment with the Hh signaling agonist SAG. The cells were analyzed by flow cytometry and stained with propidium iodide. The results of our study demonstrate that DAX1 had a significant impact on the cell cycle distribution of DU145 cells. Specifically, DAX1 significantly reduced the percentage of cells in the S phase, which was previously increased by SAG. Moreover, DAX1 increased the percentage of cells in the G1 phase (Figure 3A). These findings suggest that DAX1 induced cell cycle arrest at the G1 phase, which likely contributed to the growth inhibition in DU145 cells. Interestingly, knocking down DAX1 not only further reduced G1 arrest, which was already decreased by activating Hedgehog signaling with SAG, but also increased the proportion of cells in the S phase (Figure 3B). These findings emphasize the crucial role of DAX1 in regulating cell cycle progression, particularly in the context of Hedgehog activation. In the absence of DAX1, the disruption of regulatory mechanisms governing G1 arrest may lead to increased cell proliferation, which is initially activated by Hedgehog signaling.

### 3.4. DAX1 Suppresses the Proliferation and Growth of Prostate Cancer Cells

**Figure 4:**
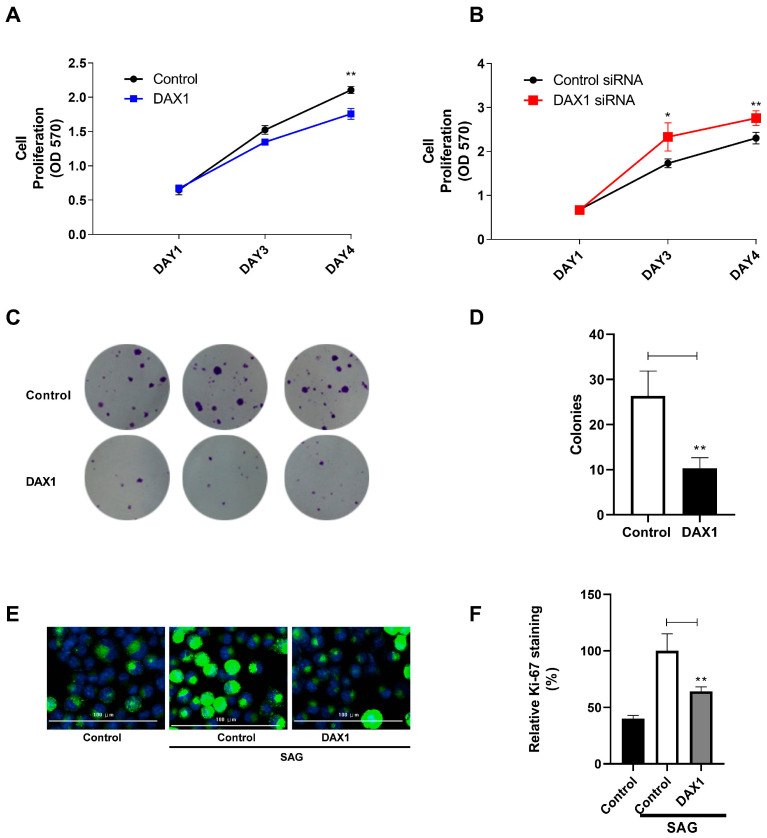
DAX1 suppresses the proliferation and growth of prostate cancer cells. (**A**) Four days of culture with 30 nM SAG were carried out on DU145 cells transduced with lenti-DAX1 or the control backbone vector. (**B**) PC3 cells treated with control or DAX1 siRNA were cultured for four days in the presence of 30 nM SAG. Proliferation was assessed by cell counting and cell survival using the MTT assay. A one-way ANOVA with Bonferroni posttest was used for statistical analysis to compare the DAX1 overexpression effect with the control. The experiment was repeated thrice. Data represent mean ± SEM; * *p* ≤ 0.05, ** *p* ≤ 0.01. (**C**) DU145 cells stably transfected with DAX1 expression lentiviral plasmids or empty vector were cultured in the presence of 30 nM SAG for 11 days and analyzed using the colony-formation assay. (**D**) The colonies stained with crystal violet are represented by a bar diagram. (**E**) Representative immune fluorescence imaging of PC3 cells transduced with lenti-DAX1 or the backbone vector control. PC3 cells were incubated with 30 nM SAG for 60 h. Green fluorescence represents Ki-67, while blue fluorescence represents nuclei stained with DAPI. Scale bar: 100 μm. (**F**) Bar diagram illustrating the percentage of cells stained positive for Ki-67 antibody. The graph compares the proportion of Ki-67-positive cells between DU145 cells transduced with lenti-DAX1 and those transduced with the backbone vector control after 60 h of incubation with 30 nM SAG. The experiment was conducted three times. Data represent mean ± SEM; ** *p* ≤ 0.01 (Student’s *t*-test, two-tailed).

To examine the impact of DAX1 on the proliferation of prostate cancer cells, an MTT assay was conducted on the DU145 cell line. Prostate cancer cells that overexpressed DAX1 exhibited inhibited cell proliferation in comparison to the control cells (Figure 4A). On the other hand, when DAX1 was knocked down, the proliferation of prostate cancer cell lines was enhanced (Figure 4B). Colony formation assays revealed that DAX1 notably decreased the colony-forming ability of DU145 cells (Figure 4C,D). To assess the impact of DAX1 on the proliferation of prostate cancer cells, we utilized the expression of Ki67 as a marker. Ki67 is a widely used marker for cell proliferation, as it is present during active phases of the cell cycle but absent in resting cells. We found that prostate cancer cells transduced with DAX1 had significantly reduced Ki67 staining compared to cells transduced with the control vector. This indicates that DAX1 inhibits prostate cancer cell proliferation (Figure 4E,F). These findings demonstrate the inhibitory effect of DAX1 overexpression on prostate cancer cell growth, particularly in the presence of Hedgehog activation.

## 4. Discussion

Increasing evidence indicates DAX1 expression in prostate cancer, breast cancer, ovary cancer, lung cancer, and Ewing’s sarcoma, although its expression pattern has shown discrepancy among different tumor types [26]. In fact, DAX1 expression in normal and prostate tumor tissues is controversial [19]. The exact function of DAX1 in prostate cancer remains unknown and needs to be investigated. DAX1 reportedly functions as a coregulatory protein rather than a transcriptional factor [13,14]. We have previously demonstrated that DAX1 controls gluconeogenesis and lipogenesis by inhibiting the transcriptional activity of hepatocyte nuclear factor 4-α and liver X receptor-α, respectively [20,27].

The Hh pathway is mediated by transcriptional factors that belong to the GLI family, whose activity is finely tuned by several molecular interactions and post-translational modifications [12]. The activity of the GLI proteins is regulated by several mitogenic and oncogenic pathways, independent of the upstream Hh pathway [8,9,10]. The identification of these regulators in cancer is an important step toward the development of effective targeted combinational therapies [28].

The findings from our experimental results provide valuable insights into the role of DAX1 in regulating the Hh pathway and its impact on cell proliferation and growth in prostate cancer cells. Both DAX1 overexpression and DAX1 knockdown experiments revealed no significant alteration in the abundance of SMO protein. This overlapping observation suggests that DAX1 specifically influences downstream components of the Hedgehog (Hh) pathway rather than directly affecting SMO protein levels. Indeed, the overexpression of DAX1 resulted in the decreased expression of the Hedgehog target genes Gli1 and Ptch1. Conversely, DAX1 knockdown experiments showed increased mRNA and protein levels of Gli1 and Ptch1. These findings indicate that DAX1 exerts regulatory control over the expression of Gli1 and Ptch1, suggesting its involvement in modulating the transcriptional activity of GLI proteins. Actually, our results demonstrate that DAX1 downregulated the transcriptional activity of GLI proteins, highlighting its role in suppressing GLI-dependent transcriptional activation. Our study also revealed the interaction between DAX1 and GLI1/GLI2 proteins. Co-immunoprecipitation assays demonstrated the physical interaction between DAX1 and GLI1/GLI2, both in exogenous overexpression systems and in endogenous protein complexes from prostate cancer cells. This interaction suggests that DAX1 may exert its inhibitory effect on the Hh pathway through interactions with GLI transcription factors.

The progression of the cell cycle, which consists of the G1, S, G2, and M phases, is the driving force behind cell proliferation. Cyclins and CDKs are the “engine of the cell cycle” for cell-cycle regulation therapies [28]. During the majority of the G1 phase, CDK4 or CDK6 associated with cyclin D1 is involved in cell-cycle progression. Thus, the cyclin D–Cdk4/6 complex promotes the progression of G1 and G1/S transition. Cyclin D1 and FOXM1, which are upregulated by Hh pathways, drive cell-cycle progression at the G1/S phase. FOXM1 induces cell cycle progression, especially at the G2/M phase, through the transcriptional upregulation of cyclin B1. These findings indicate that the Hh pathway promotes cellular progression through the induction of multiple cyclins functioning during the G1/S and G2/M phases [6,29]. Therefore, it is possible that DAX1 overexpression may contribute to decreased cyclin D1 or FOXM1 expression through the inhibition of GLI proteins. DAX1 may directly regulate cyclin D1 expression due to the presence of consensus GLI-binding site in the *CCND1* sequence and the fact that GLI1 binds to the *CCND1* promoter. As expected, we observed that DAX1 played a crucial role in regulating cell cycle progression in prostate cancer cells. The overexpression of DAX1 induced G1/S cell cycle arrest, as evidenced by a decrease in the percentage of cells in the S phase and an increase in the percentage of cells in the G1 phase. This effect was particularly significant in the context of Hh pathway activation induced by the agonist SAG. Conversely, the knockdown of DAX1 not only further reduced G1 arrest but also increased the proportion of cells in the S phase, highlighting the disruption of regulatory mechanisms governing G1 arrest in the absence of DAX1. These findings underscore the importance of DAX1 in modulating cell cycle progression and suggest that its absence may lead to increased cell proliferation, particularly in the context of Hh pathway activation.

The Hh pathway is required for the proliferation of prostate cancer cells [30]. The results obtained from the MTT assay, colony formation assay, and Ki67 immunofluorescence staining consistently demonstrate that the overexpression of DAX1 significantly reduces the growth rates of prostate cancer cells compared to the control groups. Conversely, the knockdown of DAX1 enhanced the proliferation of prostate cancer cells. These findings provide compelling evidence that DAX1 has the potential to effectively inhibit the proliferation of prostate cancer cells induced by Hh signaling.

From a therapeutic point of view, a relevant finding of this study is that DAX1 activation and Hh signaling inhibition at the level of SMO act synergistically to decrease prostate cancer cell proliferation. There has been increasing interest in the identification of proteins that target GLI proteins, the final transcription factors of the Hh pathway, which may help overcome anti-SMO resistance [13]. Hh pathway inhibitors have already been developed for human cancer therapy; however, DAX1 may be a potential target for further cancer drug development, because no drugs are currently available for clinical use [30]. Our data suggest a potential novel therapeutic approach for a subset of prostate cancer and other cancer types expressing high levels of DAX1 and with an activated Hh pathway. Indeed, targeting Hh signaling at the SMO level, with an SMO antagonist, and at the GLI1 level, with DAX1 activation, should result in a more potent inhibition of prostate cancer growth. In particular, activating DAX1 in tumors with high level of DAX1 may directly inhibit GLI1 function, resulting in a stronger inhibition of Hh signaling.

A limitation of this study is that no additional target genes for the interaction of DAX1 and GLI1/2 were identified. Using the STRING online database, the present study conducted an in silico analysis to explore potential associations. However, no significant associations were identified. The observed lack of association in the aforementioned in silico analysis may be attributed to the regulatory mechanism of DAX1, which involves binding to other transcription factors to regulate its activity, rather than binding directly to the promoter region of DNA. Another limitation of this study is the absence of in vivo or clinical samples to validate the findings. Specifically, the protein expression of DAX1 and GLI1/GLI2 in different prostatic cancer specimens should be investigated.

Furthermore, our study suggests that small molecules that activate DAX1 could be combined with existing Hh pathway inhibitors to enhance their effectiveness in treating Hh-driven tumors. By activating DAX1, these small molecules could mimic the inhibitory effects of DAX1 overexpression on Hh signaling and thus provide a more comprehensive approach to targeting Hh-driven tumors. These findings could potentially lead to the development of new drugs that target both DAX1 and the Hh pathway, improving the efficacy of current treatments for Hh-driven prostate cancer. Overall, our study provides a promising avenue for future research on the development of small molecules that activate DAX1 as a potential therapeutic approach for Hh-driven tumors.

## 5. Conclusions

In conclusion, our study highlights the critical role of DAX1 in inhibiting the Hedgehog-induced proliferation of prostate cancer cells. DAX1 inhibits the Hh pathway, interacts with GLI1 and GLI2 proteins, and induces G1/S cell cycle arrest. The disruption of DAX1 leads to the activation of the Hh pathway and the dysregulation of G1 arrest, potentially resulting in increased cell proliferation. These findings contribute to our understanding of the molecular mechanisms involved in prostate cancer progression and provide a basis for further investigation into the therapeutic potential of targeting DAX1 and the Hh pathway in prostate cancer treatment.

## Data Availability

Not applicable.

## Abbreviations

SAG	SMOOTHENED agonist
Hh	Hedgehog
GLI	glioma-associated oncogene
FOXM1	Forkhead Box M1
CCND1	cyclin D1
L32	Ribosomal protein L32
PTCH1	Patched 1
SMO	SMOOTHENED
CDK	Cyclin-dependent kinase
DAX1	dosage-sensitive sex reversal adrenal hypoplasia congenital critical region on X chromosome, gene 1
